# From biogenesis to host modulation: the expanding biology of oral streptococcal membrane vesicles

**DOI:** 10.1093/femsre/fuag049

**Published:** 2026-09-16

**Authors:** Sebastian Aguayo, Camila Leiva-Sabadini, Paula Saavedra, Davis Thalhuber, Emily Helliwell, Justin Merritt, Jens Kreth

**Affiliations:** Dentistry School, Faculty of Medicine, Pontificia Universidad Católica de Chile, Santiago, 7820436, Chile; Institute for Biological and Medical Engineering, Schools of Engineering, Medicine and Biological Sciences, Pontificia Universidad Católica de Chile, Santiago, 7820436, Chile; Dentistry School, Faculty of Medicine, Pontificia Universidad Católica de Chile, Santiago, 7820436, Chile; Institute for Biological and Medical Engineering, Schools of Engineering, Medicine and Biological Sciences, Pontificia Universidad Católica de Chile, Santiago, 7820436, Chile; Dentistry School, Faculty of Medicine, Pontificia Universidad Católica de Chile, Santiago, 7820436, Chile; Institute for Biological and Medical Engineering, Schools of Engineering, Medicine and Biological Sciences, Pontificia Universidad Católica de Chile, Santiago, 7820436, Chile; Biomaterial and Biomedical Sciences, School of Dentistry, Oregon Health & Science University (OHSU), Portland, OR 97239, United States; Biomaterial and Biomedical Sciences, School of Dentistry, Oregon Health & Science University (OHSU), Portland, OR 97239, United States; Biomaterial and Biomedical Sciences, School of Dentistry, Oregon Health & Science University (OHSU), Portland, OR 97239, United States; Department of Molecular Microbiology and Immunology, School of Medicine, Oregon Health & Science University (OHSU), Portland, OR 97239, United States; Biomaterial and Biomedical Sciences, School of Dentistry, Oregon Health & Science University (OHSU), Portland, OR 97239, United States; Department of Molecular Microbiology and Immunology, School of Medicine, Oregon Health & Science University (OHSU), Portland, OR 97239, United States

**Keywords:** biofilms, streptococci, vesicles, Gram-positives, host modulation, ecology

## Abstract

Bacterial extracellular membrane vesicles (bEVs) provide fundamental biological functions through communication, ecological adaptation, and mediation of host interactions. Gram-positive oral streptococci were long considered to be incapable of bEVs production due to their thick peptidoglycan. In contrast, recent advances in imaging, biochemical isolation, and multi-omics profiling have revealed that bEVs production is common and may represent a general trait of streptococci. This review summarizes current research on the biogenesis, cargo composition, and functional roles of bEVs, with an emphasis on oral streptococcal vesicles and their contributions to oral microbial ecology and host responses. We highlight methodological innovations for isolation and characterization of bEVs that have led to the discovery of species-specific bEVs cargo. Functionally, bEVs participate in diverse biological activities, including horizontal gene transfer, antimicrobial peptide delivery, virulence and redox modulation, and they profoundly influence host immunity by modulating cytokine signaling, epithelial barrier function, and immune pathways. Emerging evidence further suggests active roles in polymicrobial biofilm development and systemic dissemination of microbial signals relevant to health and disease. By integrating biochemical, structural, ecological, and immunological perspectives, this review provides a comprehensive overview of the current state of streptococcal and Gram-positive bEVs research and discusses its translational potential to support oral and general health.

## Teaser

This review synthesizes current advances demonstrating that Gram-positive oral streptococci actively produce extracellular membrane vesicles that mediate interbacterial communication, biofilm ecology, virulence, and host immune modulation, highlighting their biogenesis, cargo specificity, functional roles, and translational relevance to oral and systemic health.

## Introduction

The oral microbiome has emerged as one of the best characterized microbial ecosystems in the human body, due to its complexity and impact on both oral and systemic health. To date, more than 250 species have been cultured from the oral cavity (Sedghi et al. 2021), and recent advances in high-throughput sequencing and metagenomics have identified over 700 unique oral taxa (Aas et al. 2005, Kilian et al. 2016, Escapa et al. 2018). Overall, the oral microbiome is composed of prokaryotes, archaea, eukaryotes, and viruses that together establish a diverse and highly interactive biological community that inhabits its different ecological niches (Wade 2013). Furthermore, the interaction between these organisms, both cooperative and antagonistic, plays a central role in maintaining homeostasis. A specific example for corporative interactions relevant to bEVs production is the synergism between *Streptococcus sanguinis* and *Corynebacterium sp* protecting against phagocytosis (Treerat et al. 2020). In addition, hydrogen peroxide produced by *S. sanguinis* and other pyruvate oxidase (SpxB) positive oral streptococci is inhibitory to several periodontal pathogens (Herrero et al. 2016).

The oral microbiome contributes to health in distinct and well-defined ways. This includes the support and maturation of the mucosa and the immune system, participation in local nutrient metabolism, and providing colonization resistance against invading pathogens that could otherwise easily infect the host. For example, *Streptococcus salivarius* bacteriocin production may protect against Otitis Media pathogens (Chen et al. 2021). *Streptococcus oligofermentans* releases ammonia through the activity of the arginine deiminase system that is able to buffer pH fluctuations and counteract the acidogenic activity of cariogenic bacteria (Yu et al. 2020). More recently, some species of nitrate-reducing oral bacteria have been shown to potentially support cardiovascular health by generating nitric oxide, a known vasodilator and antimicrobial molecule, thus directly linking the oral microbiome to systemic physiology (Sato-Suzuki et al. 2020, Feng et al. 2023). These findings exemplify the active and dynamic roles that oral microorganisms perform to support oral health.

When this delicate ecological balance is disrupted, a state referred to as *dysbiosis*, the consequences can be detrimental to local and systemic health. Dysbiosis can be induced by poor oral hygiene, xerostomia (dry mouth), smoking, carbohydrate-rich diets, host genetics, and a variety of additional factors (Kilian et al. 2016). Importantly, dysbiosis is the principal driver of the two most prevalent oral diseases: dental caries and periodontal disease (Wade 2013, Kilian et al. 2016). Dental caries is specifically associated with an increase in the relative abundance of acid-producing and acid-tolerant species, most notably *Streptococcus mutans*. Such species generate acidic microenvironments that promote tooth demineralization (Robrish and Krichevsky 1972, De Soet et al. 2000). In addition to *S. mutans*, various other bacterial species such as *Actinomyces, Bifidibacterium, Selenomonas, Veillonella* (Aas et al. 2008) as well as fungal species mainly *Candida albicans* (Xiao et al. 2018) strongly associate with caries development as well, demonstrating the intricate ecological interactions within the oral microbiome that lead to the development of dysbiotic disease.

Similarly, periodontal disease stems from shifts in the subgingival microbiota, with Gram-negative anaerobes such as *Porphyromonas gingivalis, Treponema denticola*, and *Tannerella forsythia* initiating immune dysregulation and chronic inflammation that can ultimately lead to periodontal tissue destruction and systemic impacts upon remote tissues (Wade 2013, Baker et al. 2023). Importantly, the influence of oral dysbiosis extends well beyond the anatomical constraints of the oral cavity (Sedghi et al. 2021). Translocation of bacteria or their components into the systemic circulation has been linked to a growing list of pathologies, including Alzheimer’s disease, diabetes, cardiovascular disease, colorectal cancer, inflammatory bowel disease, rheumatoid arthritis, and obesity, reviewed in (Hajishengallis and Chavakis 2021). This evidence highlights the oral microbiome as both a sentinel of health and a potential driver of systemic diseases throughout the body.

Oral streptococci occupy a central ecological niche within the highly complex oral microbiome. As some of the earliest colonizers of the oral cavity, they represent 60%–90% of the initial microbial population on tooth surfaces within hours after cleaning (Nyvad and Kilian 1987). Even before tooth eruption, oral streptococci can adhere to mucosal surfaces like the tongue and epithelium and outcompete other microbes during initial surface attachment, reviewed in (Nobbs et al. 2009). The colonization success of streptococci lies in their extraordinary capacity for aggregation and the expression of a multitude of specialized surface proteins, known as adhesins, that allow both non-specific and specific surface attachment to tissues and the salivary pellicle. Also, they are the only genus of oral Gram-positive bacteria known to engage extensively in both intra- and inter-genus co-aggregation, enabling them to act as ecological scaffolds for biofilm development, reviewed in (Kolenbrander et al. 2002). Metagenomic analyses confirm their dominance, identifying *Streptococcus* as one of the most abundant genera in the oral cavity, followed closely by *Prevotella* (McLean et al. 2021, Manzoor et al. 2024, Oh et al. 2025). Notably, recent research has demonstrated that oral streptococci are also producers of bacterial extracellular vesicles (bEVs) (Fig. 1A), with vesicle production described in species such as *S. mutans, S. sanguinis, Streptococcus agalactiae, S. salivarius*, and *Streptococcus oralis* (Armistead et al. 2020, Kim et al. 2022, Helliwell et al. 2023, Kulig et al. 2023a, Leiva-Sabadini et al. 2025). The study of these bEVs provides a novel perspective on the role of streptococci in oral microbial ecology and host interactions.

**Figure 1:**
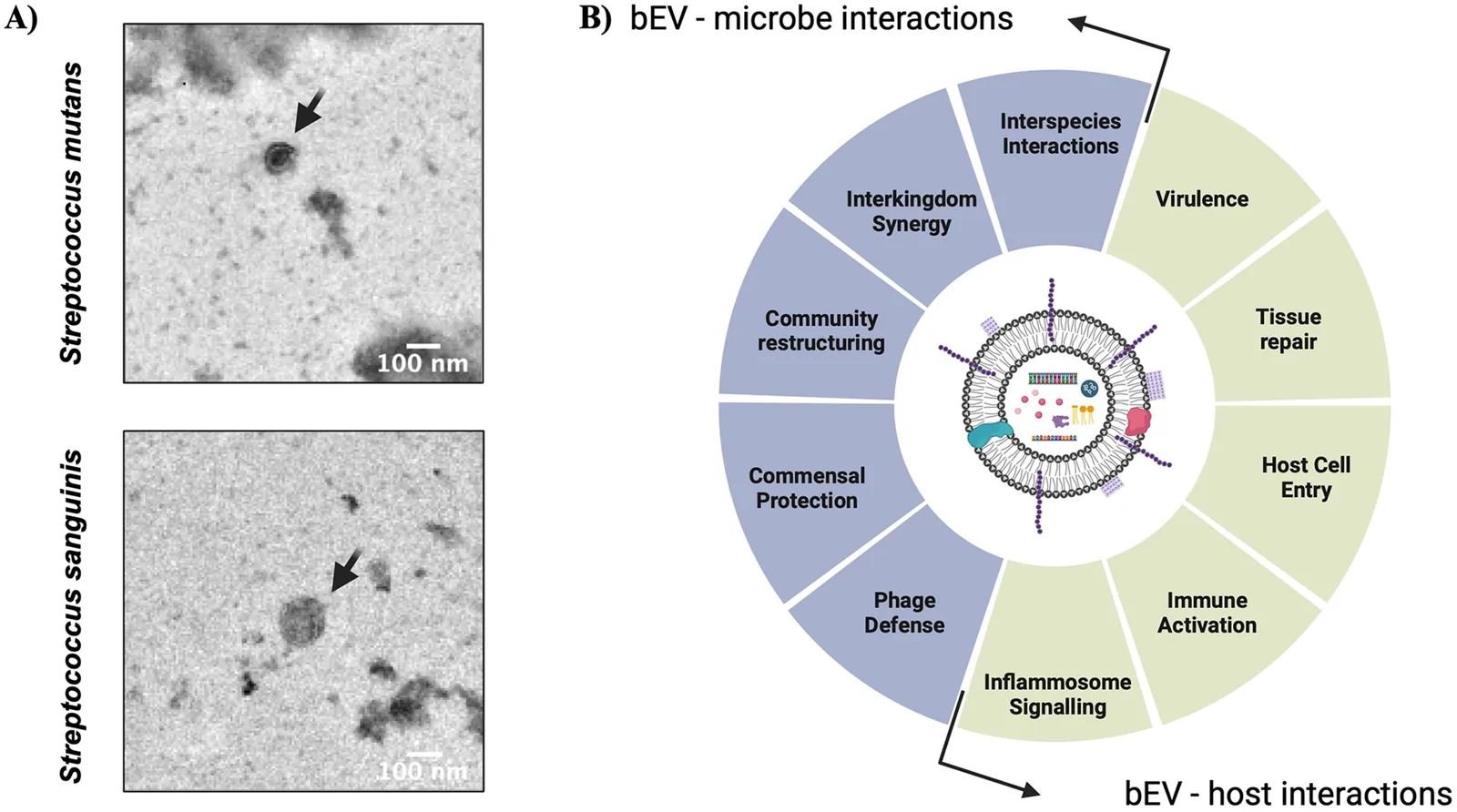
bEVs and their functional roles in Gram-positives and streptococci. (A) TEM images (negative staining) of bEV from *Streptococcus mutans* and *Streptococcus sanguinis* (unpublished). (B) Gram-positive and streptococcal bEVs participate in a wide range of biological functions that shape both host–microbe and microbe–microbe interactions. Identified roles include modulation of host immune responses, delivery of virulence and immunomodulatory factors, interbacterial signaling, nutrient acquisition and metabolic exchange, stress adaptation, and contributions to biofilm formation and spatial organization within microbial communities. These functions highlight bEVs as important mediators of communication and ecological fitness in complex host-associated environments. (Figure generated using BioRender).

The concept of extracellular vesicles (EVs) emerged in the 1970s, though their significance was long debated in the literature. Initially dismissed as cell debris, metabolic waste containers, or artifacts of cell death or laboratory methods, EVs gradually gained recognition as authentic secretory elements (Johnstone et al. 1987, Bazzan et al. 2021). Early studies with eukaryotic cells described vesicles (referred to as exosomes) in reticulocytes, where they functioned to discard obsolete components (Johnstone et al. 1987), but skepticism persisted regarding their wider biological role.

Over the past five decades, this perception has changed dramatically, and it is now well established that EVs are actively produced and secreted by virtually all cell types across all domains of life. The main functions of EVs are to serve as messengers for intercellular and inter-kingdom communication, immune modulation, tissue repair, ecological relevant interactions, and pathogenesis (Schuh et al. 2019) (Fig. 1B). Their lipid bilayer protects the internal molecular cargo. For example, the protection of DNA from hydrolysis by external DNAase treatment was initially confirmed experimentally with *Pseudomonas aeruginosa* bEVs (Kadurugamuwa and Beveridge 1995). The diverse cargo of bEVs that includes proteins, nucleic acids, lipids, antimicrobial peptides, and metabolites (Lee et al. 2009) will likely be delivered to distant or protected targets and facilitating internalization into receptor cells as demonstrated with human lung epithelial cells and *P. aeruginosa* bEVs (further reviewed in Kumar et al. 2024). In microbial communities, bEVs promote biofilm formation, mediate competitive and cooperative interactions across species, and contribute to both local and distant pathogenesis (Wu et al. 2020, Cao et al. 2026). Accordingly, bEVs have become promising candidates as diagnostic biomarkers and therapeutic tools, underscoring their relevance in both health and disease.

Thus, the purpose of this review is to discuss the latest knowledge regarding the biogenesis, composition, and function of streptococcal membrane vesicles, particularly in the context of oral ecology and disease. However, where helpful for broader context, we include examples from both Gram-positive and Gram-negative bacteria, and their systemic influence is described. To address these questions, this review follows the progression from vesicle biogenesis to cargo composition and ultimately to functional roles in microbial ecology and host interactions.

## Membrane vesicle biogenesis

### Historical perspective

In 1967, electron microscopy studies of the Gram-negative bacterium *Vibrio cholerae* revealed an excretion of cell-wall/outer-membrane material that produced membrane enclosed, sac-like structures, each narrowed at the junction between the sac and the bacterial surface. Although the authors did not use the term “membrane vesicle,” referring instead to “granules” or “sacs,” their images depict multiple stages of membrane vesicle formation. Potassium permanganate staining of the granules revealed associated fibrillar material, suggesting that the vesicular structures contain cargo (Chatterjee and Das 1967). The mechanism of bacterial extracellular vesicle (bEVs) biogenesis remains controversial, with evidence supporting both lysis-independent and lysis-dependent pathways: while *Vibrio cholerae* appears to produce bEVs through a controlled process without detectable cell lysis (Chatterjee and Das 1967), studies in *Pseudomonas aeruginosa* demonstrate that vesicle formation can result from explosive cell lysis mediated by a prophage-encoded endolysin (*lys*), highlighting fundamentally different mechanisms across species (Turnbull et al. 2016).

The Gram-negative outer-membrane “sac” formation noted above may be the first specific description of bEVs. It is also conceivable that earlier electron-microscopic studies documented bEVs production but did not recognize it as such or dismissed it as a preparation artifact. Indeed, in early images of *β*-hemolytic group A streptococci dating back to 1941 (Mudd and Lackman 1941), several photographs show cell-wall–associated structures that conspicuously resemble bEVs and fall within the size range reported in later publications.

Despite early examples of bEVs production in both Gram-positive and Gram-negative bacteria, the topic remained controversial but found an appropriate answer to major arguments raised by skeptics in the 2019 review by Coelho and Casadevall. That article refuted the main points used to argue against regulated membrane vesicle production and its biological role, effectively settling the debate (Coelho and Casadevall 2019). Even with multiple demonstrations that bEVs production is regulated and functionally purposeful (Abe et al. 2021, Krause et al. 2025, Cao et al. 2026), research on Gram-positive bEVs still lags behind work on Gram-negatives and eukaryotic systems. This gap may have been unintentionally reinforced by an influential early study on DNA as vesicle cargo (Dorward and Garon 1990). In 1990, Dorward and Garon examined 18 species, motivated by reports of DNA-containing bEVs in Neisseria gonorrhoeae (Dorward et al. 1989) and Borrelia burgdorferi (Garon et al. 1989), and concluded that “DNA is packaged within membrane-derived vesicles of Gram-negative but not Gram-positive bacteria” (Dorward and Garon 1990). Although their investigation was biased toward Gram-negatives (with 14 included species and only 4 Gram-positives), they did note occasional bEVs production by Gram-positive species Bacillus subtilis and Bacillus cereus, while reporting none for Streptococcus sanguinis or Staphylococcus aureus (Dorward and Garon 1990), two species later shown to be bEVs producers (Lee et al. 2009, Choi et al. 2018). This paper likely reinforced the assumption that the thick peptidoglycan of Gram-positives and the lack of an outer membrane preclude vesiculation.

*Staphylococcus aureus* was subsequently the first Gram-positive species reported to produce bEVs (Lee et al. 2009). Although that study did not directly demonstrate DNA cargo, its bEVs proteome included numerous proteins involved in nucleic-acid metabolism (e.g. DNA gyrase subunit A; DNA-directed RNA polymerase subunits), consistent with later reports confirming that S. aureus membrane vesicles carry both DNA and RNA (Rodriguez and Kuehn 2020). Further details about vesicle cargo are discussed later.

### Mechanisms of vesicle formation

Although this review focuses on bEVs production in streptococci (and other Gram-positives), it is useful to briefly summarize Gram-negative bEVs biogenesis to highlight key distinctions.

#### Gram-negative models

The defining difference in bEVs biogenesis between Gram-negatives and Gram-positives is determined by the bacterial cell-envelope architecture. Gram-negative bacteria have: (i) a cytoplasmic (inner) membrane enclosing the cytoplasm; (ii) a periplasm containing a very thin peptidoglycan layer (normally between 1–2 layers) (for an in depth review on peptidoglycan structure and synthesis see (Vollmer et al. 2008); and (iii) an outer membrane enriched in lipopolysaccharide (LPS), lipoproteins (e.g. Lpp), and outer-membrane proteins (Silhavy et al. 2010). This tripartite envelope strongly shapes bEVs biogenesis: most vesicles bud from the outer membrane and pinch off without compromising the inner membrane and are therefore named outer-membrane vesicles (OMV). However, in some contexts, lysis-associated events can generate vesicles that also include inner-membrane material, as shown for the aforementioned endolysin (*lys*)-mediated lysis (referred to as “cell explosion”) in *P. aeruginosa* (Turnbull et al. 2016). However, Gram-negative OMV formation is influenced by several cell-wall–associated processes and proteins (reviewed in (Nagakubo et al. 2020, Zhao et al. 2025, Zhou et al. 2025). In particular, OMV production is affected by the dissociation of anchor points between the peptidoglycan layer and the outer membrane. Several proteins serve this tethering function, cross-linking the two Gram-negative cell-wall structures. Thus, mutations that remove these anchor points create localized membrane instability and promote OMV formation. This has been shown for the widely distributed outer-membrane protein A (OmpA) and for the highly abundant lipoprotein Lpp, both serving as anchors between peptidoglycan and the outer membrane. Deletion of *ompA* or *lpp* results in hypervesiculation in several species (Valeru et al. 2014, Chen et al. 2020, Winkle et al. 2021). Accordingly, regulatory changes that alter the abundance of these proteins may allow the cell to tune OMV production.

A more robust model for membrane stress–associated production of outer membrane vesicles (OMVs) was introduced by Schertzer and Whiteley (Schertzer and Whiteley 2012) and has recently been further validated experimentally. The bilayer-couple model is based on the insertion of the *P. aeruginosa* quorum-sensing molecule 2-heptyl-3-hydroxy-4-quinolone (PQS) into the outer leaflet of the outer membrane. This asymmetric incorporation of PQS leaves the inner leaflet largely undisturbed, resulting in an expansion of the outer leaflet surface area relative to the inner leaflet. Consequently, the outer membrane bulges outward, leading to OMV budding to accommodate the increased surface area. Importantly, this process preserves the integrity of the underlying peptidoglycan layer, supporting a lysis-independent mechanism of OMV formation in Gram-negative bacteria. Notably, the bilayer-couple model is not unique to *P. aeruginosa* in response to PQS, as PQS can also induce OMV production in other species. Furthermore, other bacteria secrete small molecules capable of triggering OMV formation, suggesting that small-molecule–induced membrane curvature may represent a broadly conserved mechanism of OMV biogenesis (Horspool and Schertzer 2018). In addition to small-molecule–mediated effects, periplasmic stress can also promote OMV formation. Accumulation of periplasmic macromolecules, such as peptidoglycan fragments, creates localized pressure points that drive budding through increased turgor pressure (Furuyama and Sircili 2021), as shown for an autolysin mutant of *P. gingivalis* (Hayashi et al. 2002). Likewise, changes in LPS charge and remodeling, as well as specific lipids such as cardiolipin, can modulate curvature of the outer membrane. Curved membrane areas have been suggested to influence vesicle formation (Beltrán-Heredia et al. 2019, Kim et al. 2026).

Taken together, OMV production in Gram-negatives is not controlled by a single gene or process. It is unlikely that a species simply represses OmpA or overexpresses autolysins to “cause” vesiculation; rather, bacteria integrate multiple envelope cues such as tether abundance, periplasmic stress, and LPS remodeling to fine-tune vesicle production. This multifactorial control parallels Gram-positive membrane vesicle production, which likewise lacks a single master regulator for vesiculation as discussed below.

#### Gram-positive vesicle biogenesis in streptococci

The cell wall architecture of streptococci and other Gram-positive bacteria differs from that of Gram-negatives in that it lacks an outer membrane layer (Rohde 2019). Streptococcal membrane vesicles therefore originate from the cytoplasmic membrane and, unlike outer membrane vesicles of Gram-negative bacteria, must ultimately traverse the thick peptidoglycan layer to reach the extracellular environment. Whether this process occurs exclusively through lysis-dependent mechanisms or can also occur in the absence of complete cell lysis remains an open question. If vesicles are released without complete cellular lysis, passage through the peptidoglycan would represent a substantial physical challenge. Streptococcal bEVs typically range from 80 to 200 nm in diameter (Codemo et al. 2018, McCutcheon et al. 2021, Helliwell et al. 2023), whereas the peptidoglycan layer of live *S. agalactiae* measures ∼30 nm in thickness (Saar Dover et al. 2015), a value likely representative of other streptococci. In comparison, other Gram-positive bacteria can possess peptidoglycan layers approaching 100 nm in thickness (Rohde 2019) consisting of multiple layers of a mesh-like structure that could impede vesicle passage. The effective pore size of Gram-positive peptidoglycan has been estimated to be ∼2.1 nm in *B. subtilis* (Demchick and Koch 1996) although larger surface-facing pores, reaching up to 60 nm in diameter and 23 nm in depth, have been visualized in living *S. aureus* cells by atomic force microscopy (Pasquina-Lemonche et al. 2020). Under physiological conditions, the peptidoglycan behaves as a hydrated, porous, gel-like network with a degree of structural flexibility. However, the region immediately adjacent to the cytoplasmic membrane appears substantially more compact, with pores sufficiently small to prevent membrane protrusion (Pasquina-Lemonche et al. 2020). These structural observations suggest that the release of intact vesicles through an undisturbed cell wall would be difficult. Consequently, if non-lytic vesicle release occurs, it would likely require localized remodeling or transient degradation of the peptidoglycan. This process carries the risk of cell lysis when openings through the entire peptidoglycan layer exceed 15 to 24 nm in width (Mitchell et al. 2013). However, localized hydrolysis is a normal component of peptidoglycan synthesis and turnover during bacterial growth and division, although direct evidence demonstrating that these remodeling events permit membrane vesicle passage is currently lacking. Therefore, bEVs release involving localized peptidoglycan degradation must be tightly regulated and is likely coupled to mechanisms of peptidoglycan turnover and homeostasis.

Several observations nevertheless suggest a relationship between peptidoglycan remodeling and bEV production (specific genetic pathways related to peptidoglycan remodeling involved in bEVs biogenesis are discussed in the following section 2.4). In *Streptococcus pyogenes*, sublethal concentrations of penicillin, which reduce peptidoglycan cross-linking, increase vesicle production (Biagini et al. 2015). Similarly, *S. aureus* mutants lacking penicillin-binding protein 4 (Pbp4), which exhibit reduced peptidoglycan cross-linking, also produce increased numbers of membrane vesicles, whereas deletion of the daughter cell separation autolysin Sle1 significantly reduces vesicle production (Wang et al. 2018). It should be noted, however, that *β*-lactam treatment, defects in cell wall synthesis or changes in autolysin activity may also result in some levels of cell lysis, and these studies therefore do not distinguish between vesicle release through localized cell wall remodeling and lysis-dependent mechanisms. In *S. mutans*, the competence-regulated autolysin LytF has also been implicated in vesicle production. Competence induction results in LytF-dependent lysis of a subpopulation of cells, and deletion of *lytF* significantly reduces vesicle production (Lemme et al. 2011, Son et al. 2012, Nagasawa et al. 2025) (Fig. 2). Likewise, the major autolysin AtlA contributes to vesicle production in *S. mutans* (Morales-Aparicio et al. 2020) while several prophage-independent autolysins have been implicated in membrane vesicle biogenesis in *B. subtilis* (Abe et al. 2021). Collectively, these findings establish a close association between autolysin activity, cell wall remodeling, and vesicle production. However, whether membrane vesicles are released predominantly through localized peptidoglycan remodeling without complete cell lysis, through controlled lysis of a subset of cells, or through a combination of both mechanisms remains unresolved and will require direct experimental investigation (Fig. 2).

**Figure 2:**
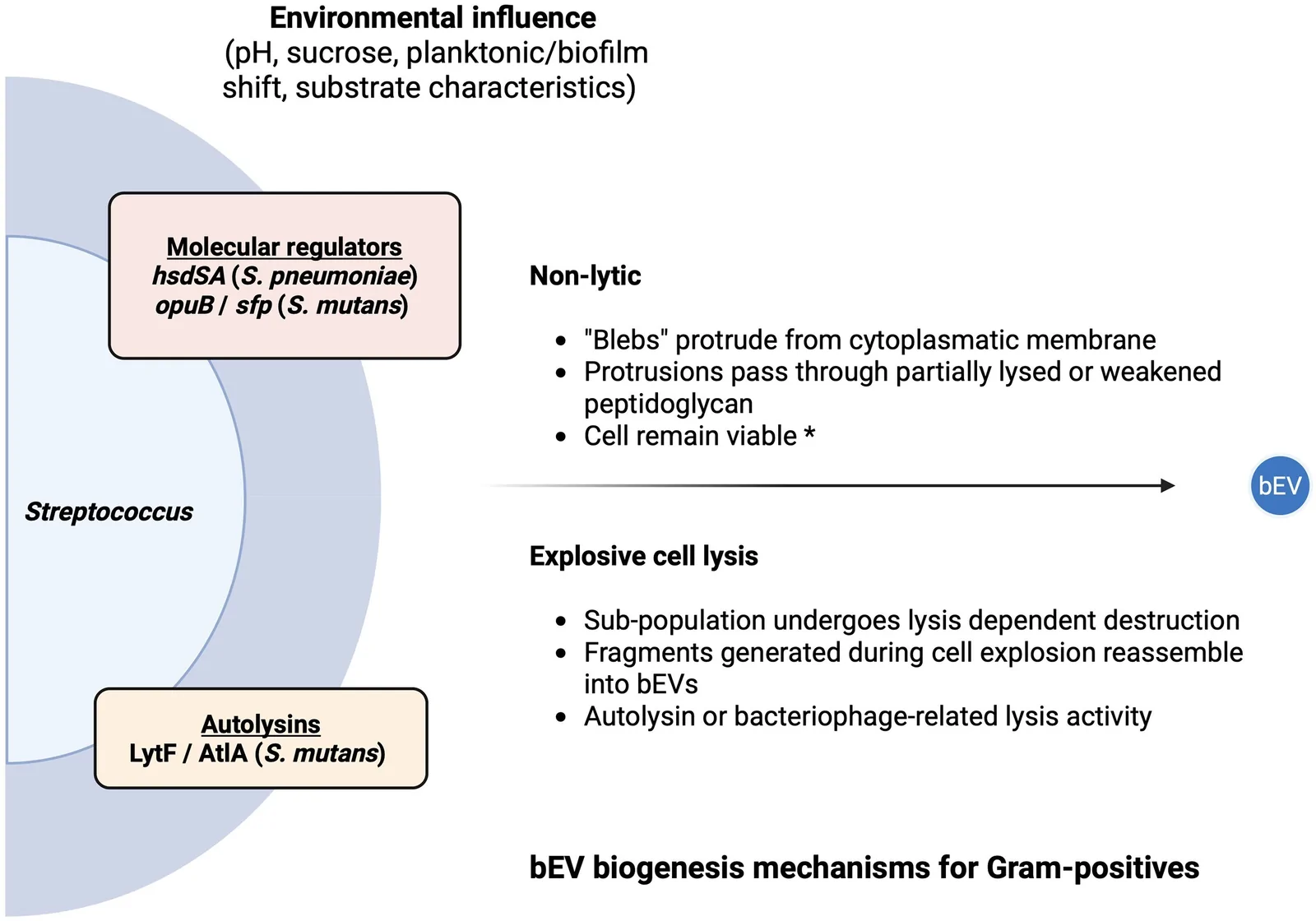
Biogenesis of streptococcal bEVs. Proposed mechanisms of bEV biogenesis in Gram-positive bacteria. Current evidence supports at least two routes of vesicle formation: (i) non-lytic membrane blebbing, in which membrane protrusions are released from viable cells, and (ii) explosive cell lysis, where a subpopulation of cells undergoes autolysin-mediated cell wall degradation and membrane fragmentation, generating vesicular structures. Multiple cellular pathways and molecular regulators as well as autolysins have been implicated in bEV production. * Non-lytic vesicle formation has primarily been visualized using STORM microscopy on fixed cells (Jeong et al. 2022); therefore, these observations demonstrate vesicle budding from apparently intact cells but do not exclude the possibility of subsequent cell lysis at later stages. (Figure generated using BioRender).

### Molecular and cellular processes driving vesiculation

Microscopic imaging has been central both to the initial discovery of bacterial extracellular vesicles (bEVs) (Chatterjee and Das 1967) and to current mechanistic understanding of their biogenesis. Scanning electron microscopy (SEM) has visualized discrete regions of membrane protrusion on the surface of *S. agalactiae* and *S. pneumoniae*, consistent with conserved vesicular budding mechanisms (Surve et al. 2016, Mehanny et al. 2020). In *S. mutans*, vesicle formation occurs across multiple growth stages, including planktonic, surface-attached, and biofilm conditions, and appears to arise from non-uniform sites across the cell surface, resembling patterns observed in *B. subtilis* (Brown et al. 2014, Leiva-Sabadini et al. 2025). High-resolution imaging studies in *S. aureus* and *S. epidermidis* indicate that extracellular vesicle biogenesis is a highly regulated, sequential process initiated by controlled cytoplasmic membrane blebbing and followed by localized peptidoglycan remodeling that enables vesicle release. Using multicolor Stochastic Optical Reconstruction Microscopy (STORM), Jeong et al. directly visualized membrane protrusions that expanded and constricted prior to detachment from morphologically intact cells, providing compelling structural evidence that Gram-positive bacteria can generate vesicles through localized cell wall remodeling without requiring complete cell lysis (Jeong et al. 2022). These new formed vesicles were often retained within the so-called “inner wall zone,” suggesting a precursor state that requires subsequent localized peptidoglycan degradation for final release. Notably, increased vesicle production was observed in dividing cells, particularly during active septum formation, supporting a strong link between vesiculogenesis and cell wall remodeling processes associated with growth and division (Jeong et al. 2022). Furthermore, the investigation by Jeong et al. provides strong evidence that Gram-positive bacteria can generate extracellular vesicles through at least three distinct pathways, including two membrane blebbing mechanisms associated with localized peptidoglycan remodeling and one pathway involving explosive cell lysis. While autolysin activity is likely essential for creating localized sites permissive for vesicle release, the available data do not establish that all autolysin-dependent vesicle formation is a consequence of cell lysis, supporting the coexistence of both lytic and non-lytic mechanisms. Although the study provides compelling structural evidence for non-lytic bEV biogenesis, it does not definitively demonstrate sustained cellular integrity following vesicle release. STORM microscopy captures only static snapshots, effectively freezing cells at a single moment in time, and therefore cannot determine the subsequent fate of individual cells. Live-cell imaging would provide a more powerful mechanistic approach by enabling tracking of vesicle-producing cells over time to determine whether membrane blebbing and non-lytic vesicle release represent non-lytic events or merely the initial stages of a larger cell lysis process that occurs later. Thus, while these imaging-based observations support a model in which bEV formation is a coordinated, dynamic process tightly coupled to fundamental aspects of Gram-positive cell envelope physiology, the relative contributions of lytic and non-lytic pathways remain unresolved.

Although evidence supporting non-lytic mechanisms associated with bEV production in Gram-positive bacteria is available, this topic remains controversial. The following sections outline potential genetic determinants implicated in streptococcal bEV production that are consistent with non-lytic pathways and therefore warrant further experimental validation to help resolve this controversy.

### Genetic determinants of vesicle production

While there is likely no single master regulator of bEVs production in streptococci (and Gram-positives), many genes appear to influence bEVs biogenesis. To explore the extent of genetic influence upon bEVs production in *S. sanguinis*, Helliwellet al. performed a Pheno-RNA transcriptomic analysis comparing multiple growth conditions known to experimentally affect bEVs production (Helliwell et al. 2023). Pheno-RNA enables correlation of gene expression levels across several conditions to identify candidate genes that may regulate a given phenotype (Darwiche and Struhl 2020). In total, 46 genes were found to be significantly associated with bEVs production under the selected growth conditions, either up- or downregulated (Helliwell et al. 2023). While this set is presumably not exhaustive (as it reflects only the specific environmental growth conditions tested), several key candidate genes were noted. For example, two *fmtA*-like genes were upregulated under bEV-producing conditions. In *S. aureus*, FmtA interacts with wall teichoic acids and contributes to cell envelope architecture and peptidoglycan homeostasis (Rahman et al. 2016). In addition, teichoic acids have been identified as components of bEVs produced by *Limosilactobacillus antri* (Yamasaki-Yashiki et al. 2024). While these observations suggest that cell wall-associated polymers may influence bEV biogenesis, their precise mechanistic role remains unclear. Alterations in teichoic acid metabolism can compromise cell envelope integrity and may promote vesicle production indirectly through cell envelope stress or lysis. Therefore, whether FmtA-dependent remodeling contributes directly to vesicle release or reflects secondary consequences of altered cell envelope physiology remains to be determined. Furthermore, downregulation of these genes may reduce peptidoglycan synthesis or otherwise decrease cell wall rigidity, thereby facilitating bEVs release (Helliwell et al. 2023), supporting the idea that vesiculation in streptococci is closely tied to cell wall remodeling and peptidoglycan abundance. A connection between peptidoglycan synthesis and bEVs production has also been demonstrated in *Streptococcus pneumoniae* (Wang et al. 2024). The phase-variation gene *hsdS_A_* appears to regulate bEVs biogenesis by modulating the mRNA expression of peptidoglycan synthesis–related genes. Compared to the wild type, the *hsdS_A_* mutant exhibited a significantly thicker cell wall with nearly a two-fold increase in peptidoglycan content, driven by elevated expression of major genes involved in cell wall biosynthesis. Consequently, the mutant produced and secreted fewer bEVs into the environment. Notably, membrane vesicle cargo was also altered: the bEVs from the *hsdS_A_* mutant contained reduced levels of the key pneumococcal virulence factor pneumolysin (Wang et al. 2024). This compositional change further influenced host interactions with pneumococcal vesicles, a topic discussed in a later section.

Additional genes involved in bEV production have also been identified in *S. mutans*. As a possible adaptation to osmotic stress, which is a common environmental challenge in the oral cavity, *S. mutans* may regulate bEV biogenesis through the activity of the OpuB ATP-binding cassette (ABC) transporter (Wang et al. 2025). In *B. subtilis*, OpuB transports compatible solutes in response to elevated osmotic pressure (Kappes et al. 1999), although this function has not yet been experimentally confirmed in *S. mutans*. Deletion of *opuB* resulted in significantly increased production of smaller bEVs under neutral pH conditions together with marked alterations in vesicle phospholipid and fatty acid composition (Wang et al. 2025). Under acidic conditions, total vesicle production was comparable to the wild type, whereas differences in vesicle lipid composition became even more pronounced. These findings demonstrate that OpuB influences vesicle production and cargo composition; however, the underlying mechanism remains unresolved. One possible interpretation, which we propose here as a hypothesis, is that OpuB-dependent osmotic sensing contributes to anticipatory membrane remodeling. In the oral cavity, elevated osmolarity following carbohydrate intake commonly precedes the subsequent decrease in pH caused by bacterial fermentation. Under this scenario, osmotic stress could serve as an early environmental cue that initiates membrane remodeling before acidification occurs, with the resulting changes in membrane lipid composition being reflected in the lipid composition of released bEVs. This hypothesis is speculative and remains to be experimentally tested.

The lipidomic profile of bEVs is further influenced by the *sfp* gene in *S. mutans* (Morales-Aparicio et al. 2020, Wen et al. 2021). This gene is responsible for the biosynthesis of the lipopetide surfactin in *B. subtilis* (Nakano et al. 1988) and has a homolog in *S. mutans* (Morales-Aparicio et al. 2020). Deletion of *sfp* leads to a pronounced hypervesiculation phenotype. Notably, this increase in bEVs output is accompanied by a dramatic shift in lipid composition: vesicles from the *sfp* mutant exclusively contain polyunsaturated fatty acids (Morales-Aparicio et al. 2020, Wen et al. 2021). Although the precise mechanism remains unclear, changes in fatty acid composition alter membrane biophysical properties, such as fluidity, curvature, or tension, which in turn could facilitate or enhance bEVs formation as has been demonstrated in other bacterial species (Mashburn-Warren et al. 2008, Wang et al. 2018).

In summary, while certain differences in vesicle biogenesis exist between Gram-positives and Gram-negatives, the process of vesiculation and secretion of bEVs into the environment is broadly conserved. Vesicle production is generally considered an evolutionarily ancient process, as it has been observed across all domains of life (Gill and Forterre 2016, Ghanam et al. 2022), suggesting that all microbes either produce or are capable of producing membrane vesicles. Assuming vesiculation arose prior to the evolution of complex cellular architecture, it is conceivable that vesicle production is ancestrally linked to fundamental processes such as daughter cell formation and is therefore indispensable for life. It is thus not surprising that nucleic acids are commonly found as part of vesicle cargo (Nakano et al. 1988, Joshi et al. 2021), highlighting their role in horizontal gene transfer and microbial evolution. This function likely demands regulatory plasticity to allow adaptation to changing environmental conditions, further supporting the idea that no single master regulator controls vesicle biogenesis. Instead, cellular physiology seems to be the principal regulator. While these mechanisms define how vesicles are formed, their biological impact is ultimately determined by their molecular cargo (Fig. 3A), which varies dynamically with environmental conditions and bacterial physiology. Understanding bEVs composition is therefore essential, as selective cargo loading determines their roles in microbial communication, ecological adaptation, and host modulation. However, accurate interpretation of vesicle function is closely linked to the methods used for their isolation and characterization. Differences in isolation strategies can influence vesicle yield, purity, and apparent cargo composition, shaping conclusions about their biological roles. This is particularly relevant for Gram-positive systems, where lower vesicle abundance and the structural limitations of the thick peptidoglycan layer complicate recovery. In complex environments such as the oral microbiome, vesicles derive from multiple microbial and host sources, further emphasizing the need for carefully controlled approaches. Thus, methodological considerations are central to understanding streptococcal bEVs biology and distinguishing true biological variation from potential experimental artifacts.

**Figure 3:**
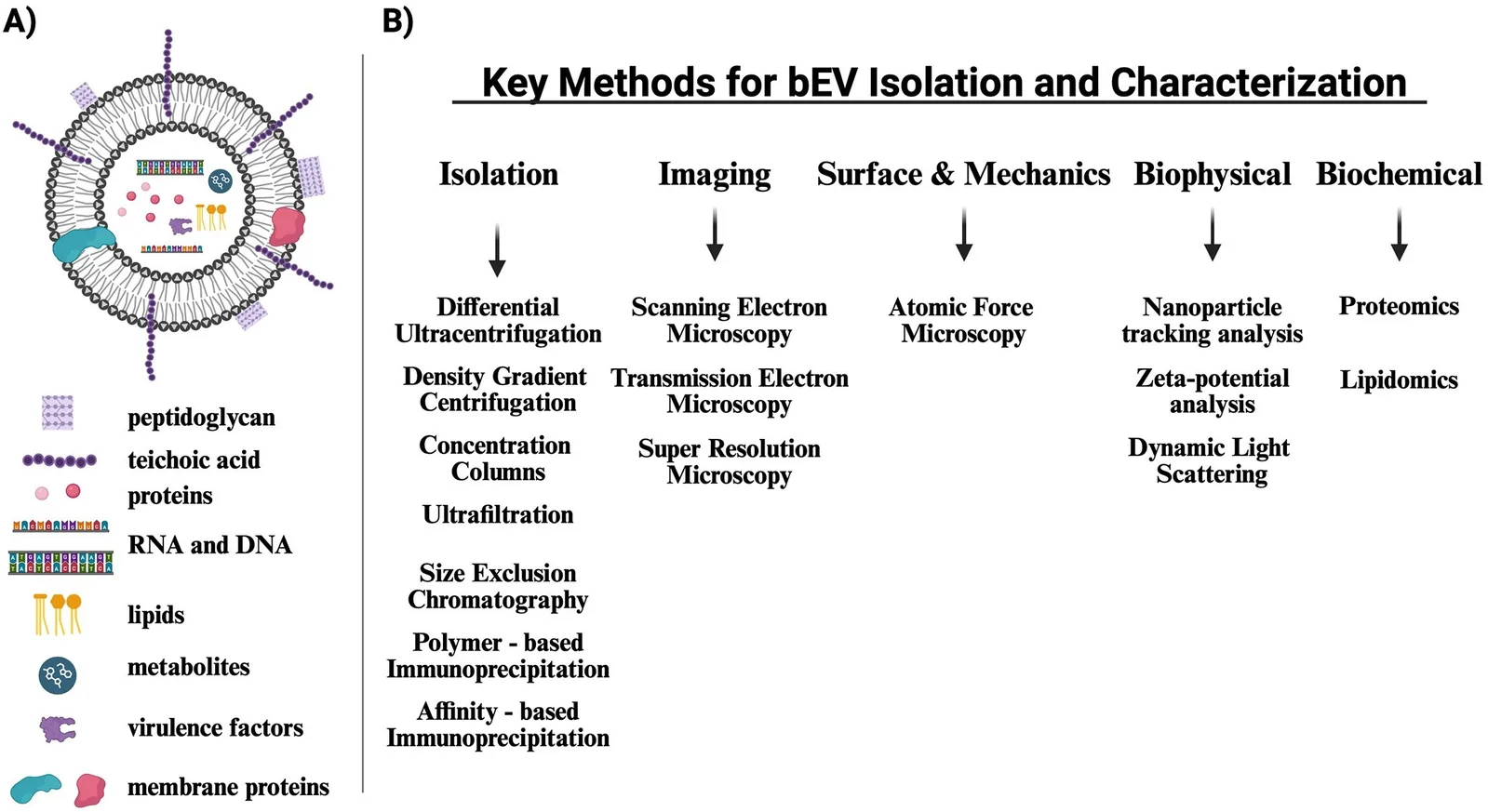
Gram-positive bEVs: structure, isolation, and characterization. (A) General overview of Gram-positive cell envelope architecture and bEV cargo. Gram-positive bEV cargo has been extensively characterized and comprises a diverse array of cell-derived biomolecules, including peptidoglycan fragments, teichoic acids, nucleic acids (RNA and DNA), proteins, lipids, and metabolites. (B) Key methods for bEV isolation and characterization that have been instrumental in the discovery of bEVs and in elucidating their biological functions. (Figure generated using BioRender).

## Methodological considerations in bEVs research.

While the initial description of bEVs in *V. cholerae* relied on electron microscopy to provide visual proof of their existence, a wide range of biochemical and biophysical techniques are now essential for their characterization (Fig. 3B). These approaches have been critical for linking vesicle biogenesis to function and for uncovering cargo variability across species and environmental conditions. For detailed protocols, readers are referred to recent methodological reviews (Tiwari et al. 2021, Welsh et al. 2024, Dai et al. 2025). Importantly, methodological choices strongly influence vesicle yield, purity, and apparent composition, making them central to interpreting biological function, particularly in Gram-positive systems where vesicle recovery remains challenging.

### Isolation of streptococcal bEVs

Ultracentrifugation remains the most widely used technique for bEVs isolation and is typically combined with additional centrifugation-based steps to improve yield and purity (Gardiner et al. 2016). Standard workflows include removal of cells and debris, filtration (commonly through 0.22 μm filter), and high-speed pelleting. Vesicle-compatible buffers such as PBS or HEPES are commonly used, while harsh detergents are avoided to preserve vesicle integrity. Density gradient ultracentrifugation, particularly employing iodixanol gradients (10%–40%), is frequently applied to separate vesicles based on buoyant density and reduce contamination, and has been successfully used in *S. pneumoniae* and *S. mutans* (Morales-Aparicio et al. 2020, Cui et al. 2024). These approaches also enable partial separation of vesicle subpopulations.

Because streptococcal bEVs yields are often low, large culture volumes are required. Volume-reduction methods, such as concentration columns, facilitate sample handling and have been used in *S. sanguinis* and *S. agalactiae* (Surve et al. 2016, Armistead et al. 2020, Helliwell et al. 2023), although they introduce additional cost.

Alternative methods include ultrafiltration, which enables rapid and efficient size-based separation while avoiding high shear forces that potentially can compromise bEVs (Welsh et al. 2024, Dai et al. 2025). However, smaller non-vesicular particles may co-isolate, and vesicle loss due to membrane adhesion has been reported (Gardiner et al. 2016). Size exclusion chromatography provides gentle and reproducible fractionation and is often used as a final step for purification (Corso et al. 2017), whereas polymer-based precipitation is scalable but prone to co-precipitation of contaminants (Pons Royo and Jungbauer 2025).

A major challenge remains the isolation of species-specific vesicles from complex microbial environments such as the oral cavity (Sedghi et al. 2021, Şenel 2021), where mixed populations from microbial and host sources complicate analysis. Affinity-based immunoprecipitation targeting species-specific epitopes or conserved Gram-positive markers such as lipoteichoic acids represents a promising strategy (Alves et al. 2017, Okamoto et al. 2025, Velimirov and Velimirov 2024). Engineered systems using epitope-tagged vesicle proteins may further enable selective isolation in controlled *in vitro* polymicrobial models.

### Imaging and biophysical characterization

Imaging techniques remain fundamental for studying vesicle biogenesis, morphology, and size. SEM has visualized membrane blebbing in *S. agalactiae* and *S. pneumoniae*, while *S. mutans* displays vesicle formation across growth states without defined budding sites (Brown et al. 2014, Surve et al. 2016, Mehanny et al. 2020, Leiva-Sabadini et al. 2025). TEM has been used in general to confirm spherical morphology and size heterogeneity, and has demonstrated surface-associated virulence factors, such as M protein, on bEVs (Liao et al. 2014, Pan et al. 2023). Advanced imaging approaches, including ultrathin sectioning and STORM microscopy, have further shown that vesicle release in Gram-positive bacteria coincides with localized cell wall remodeling (Wang et al. 2018, Jeong et al. 2022).

Atomic force microscopy (AFM) complements optical imaging by enabling high-resolution surface analysis under near-physiological conditions (Dufrêne 2008, James et al. 2017). Although underutilized for streptococcal vesicles, AFM studies have demonstrated growth-dependent changes in vesicle size and morphology and revealed links between vesicle composition and environmental conditions (Surve et al. 2016, Leiva-Sabadini et al. 2025, Askarian et al. 2018). AFM has also been applied to assess host tissue responses to vesicle exposure, highlighting its broader utility (Parisse et al. 2017).

Biophysical characterization is commonly performed using nanoparticle tracking analysis (NTA), dynamic light scattering (DLS), and *zeta* potential measurements (Kowkabany et al. 2024, Welsh et al. 2024). NTA allows simultaneous particle sizing and quantification, while DLS provides information on size distribution and heterogeneity (Stetefeld et al. 2016). *Zeta* potential reflects vesicle surface charge, which is typically negative in Gram-positive systems due to phospholipids and teichoic acids (Midekessa et al. 2020). These measurements are essential for comparing vesicle populations and linking physical properties to functional outcomes, although they remain sensitive to experimental conditions and contaminants.

### Biochemical and omics characterization

Mass spectrometry-based proteomics remains the primary tool for characterizing streptococcal bEVs cargo but is highly sensitive to growth conditions, strain variability, and isolation workflows. Proteome profiles can vary significantly between biological replicates and across strains (Olaya-Abril et al. 2021, Helliwell et al. 2023, Cui et al. 2024), emphasizing the need for standardized experimental design and transparent reporting.

Lipidomic analyses, typically combining thin-layer chromatography and LC–MS/MS, have revealed selective lipid enrichment in bEVs compared to the parent membrane, supporting controlled vesicle formation rather than nonspecific shedding (Biagini et al. 2015, Morales-Aparicio et al. 2020). Similarly, metabolomic studies have identified condition-dependent cargo changes linked to bacterial metabolic adaptation (Cao et al. 2020). Together, these approaches demonstrate that streptococcal bEVs carry selectively packaged and biologically relevant cargo. At the same time, they demonstrate that experimental variables can strongly influence observed vesicle composition. Thus, consistent methodology, controlled conditions, and careful interpretation are essential to distinguish genuine biological variation from methodological artifacts and to place vesicle function into its broader ecological and host-associated context. These methodological advances have enabled detailed characterization of vesicle cargo, revealing that streptococcal bEVs carry highly dynamic molecular content that shape their biological functions.

## Molecular cargo and functional properties of streptococcal bEVs.

The biological activity of bEVs is determined by their cargo (Fig. 3A), which reflects both species-specific traits and environmental conditions. These molecular cargos are dynamic and can vary depending upon bacterial physiology and the context under which the bEVs were produced. For instance, *S. mutans* produces bEVs with markedly different proteomic profiles depending upon whether the bacteria grow in planktonic form or within a biofilm (Leiva-Sabadini et al. 2025). Biofilm-derived bEVs have been found to be enriched in virulence-associated proteins, suggesting that vesicle production is adapted to the streptococcal ecological context. Similarly, environmental stressors, such as pH, can profoundly alter vesicular content. *S. mutans* bEVs released at pH 7.5 vs. pH 5.5 to model health vs. cariogenic conditions showed that not only the size of bEVs varies with pH, with a significant decrease in vesicle diameter at low pH, but also that each condition has unique proteins loaded as vesicle cargo (Cao et al. 2020).

### Proteins

Across strains of oral streptococci, bEVs are highly abundant in protein content and selectively loaded with content that can promote biofilm formation and interspecies interactions. Most bEVs omics studies report hundreds of distinct streptococcal proteins as cargo, with content variations as a function of strain genetics and environmental conditions. In a study conducted by Cao et al (2020) 344 shared proteins were detected under both pH values, pH 7.5 had 151 unique proteins and pH 5.5 had 7 unique proteins. Several proteins also appeared at higher abundance under low pH, including the glucosyltransferases GtfB and GtfD. GtfB is essential for *S. mutans* sucrose-dependent biofilm formation and cariogenicity (Cao et al. 2020). Investigations into the lineage-specific functions of different *S. agalactiae* strains showed that production and composition of their bEVs vary. Exemplified by serotype ST-1 strains, which had fewer unique proteins compared to several other serotypes, the total number of identified proteins ranged between 239 and 614 per strain, demonstrating considerable variation. Detailed analysis of the proteins with regard to virulence factors demonstrated that several purported virulence factors, including C5a peptidase, hyaluronidase, and sialidase, were highly enriched depending on the serotype (McCutcheon et al. 2021), similar to reported differences in virulence-factor cargo of *S. pneumoniae* (Battista et al. 2023), and likely a common theme among streptococci. In *S. sanguinis*, research has shown that the composition of bEVs shifts with carbohydrate availability, showing distinct proteomic signatures when cultured with glucose versus sucrose (Helliwell et al. 2023). Overall, this plasticity in bEVs cargo and composition as a function of environmental conditions highlights the dynamic nature of vesicle-mediated communication. Most importantly, by tailoring their cargo to specific environments, streptococcal bEVs serve as dynamic tools for survival, competition, and ecological success in the oral cavity.

In *S. mutans*, glucosyltransferases (GtfB/GtfC) can be consistently found as abundant vesicle cargo and remain biologically active to interact with target cells and promote biofilm formation (Morales-Aparicio et al. 2020, Nakamura et al. 2020, Wu et al. 2020). bEVs also carry adhesion-associated proteins such as *S. mutans* GbpB, wall-modifying enzymes such as the autolysin AtlA, and secretion machinery molecules, which is consistent with the presence of selective bEVs secretion routes instead of random vesiculogenesis (Morales-Aparicio et al. 2020). Recent reports have also found thiol peroxydase (Tpx), the molecular chaperones GroES and GroEL, thioredoxin reductase (TrxB), and S-ribosylhomocysteine (LuxS), among others, as cargo in *S. mutans* bEVs, all of which are virulence-related proteins with well described functionality in biofilm formation, oxidative stress resistance, and signaling (Leiva-Sabadini et al. 2025). The conserved presence of the AtlA autolysin across streptococcal bEVs hints that its peptidoglycan-degrading activity may be directly involved with vesiculogenesis (Ahn and Burne 2006, Morales-Aparicio et al. 2020). Furthermore, proteomic surveys of Mitis-group streptococci similarly found bEVs enriched for cytoplasmic and membrane-associated proteins implicated in bacterial attachment, competition, biofilm formation, and stress tolerance, supporting the idea that oral streptococci derived bEVs are active carriers of functional protein cargo involved in microbe-microbe and host-pathogen interactions (Matsumoto et al. 2025).

### Nucleic acids

Alongside proteins, DNA, micro RNAs (miRNAs), and other nucleic acids are commonly found as cargo in EVs. Although the presence of nucleic acids in oral streptococcal bEVs is less explored, it is known that *S. mutans* packages extracellular DNA (eDNA) into vesicles during early exponential phase and overnight growth (Liao et al. 2014). Furthermore, bEVs production or eDNA amounts were not altered in sortase (SrtA) deficient mutant cells, although changes in protein cargo profiles were observed (Liao et al. 2014), hinting towards differential modes of regulation for vesicle nucleic acid content. *Streptococcus mutans* eDNA has also been found to bind to bEVs and promote biofilm formation across many oral streptococci (Senpuku et al. 2019). Thus, bEVs are believed to be an active mechanism for eDNA release by *S. mutans*, particularly in response to decreased glucan presence in the surrounding biofilm environment (Rainey et al. 2019). Similarly, EV-mediated horizontal gene transfer has been demonstrated in *S. pneumoniae*, as pneumococcal bEVs have been found to carry surface-associated DNA that is delivered to recipient cells, thereby promoting transformation and spread of resistance alleles (Lass et al. 2023). These experiments validate streptococcal EVs as vectors for DNA exchange and suggest that oral streptococci could potentially use similar routes within polymicrobial biofilms. Additionally, recent work has demonstrated that *S. mutans* EVs also transport small RNA cargo that include tRNA-derived molecules with biological activity in host cells (Oh et al. 2025), particularly as TLR-3 receptor ligands in the context of epithelial wound healing (Oh et al. 2025).

### Lipids and metabolites

Similar to protein and nucleic acid cargo, bEVs lipidomes can present varied composition and abundance profiles as a function of the metabolic state or environmental conditions in which they were produced. The *S. mutans* bEVs lipidome is dominated by long-chain fatty acids containing up to 80 carbons that include saturated, monounsaturated, and polyunsaturated lipids (Morales-Aparicio et al. 2020). The proportion between these constituents was different in bEVs derived from sortase-deficient *S. mutans* strains, and are believed to determine bEVs curvature, size, and biological properties (Morales-Aparicio et al. 2020). In oral streptococci, lipoproteins and lipoteichoic acid (LTA) are crucial bioactive lipid-associated molecules that can be transported in bEVs (Moghaddam et al. 2025) and activate host immune cells and osteoclastogenesis in the context of periodontal disease (Kim et al. 2022). Lipidome studies in streptococci have been pivotal for understanding both bEVs biogenesis and virulence, and often provide broader insights into streptococcal membrane organization, since bEVs lipid profiles are routinely compared to those of the parent cell membrane. For *S. pyogenes*, combined thin layer chromatography and LC–MS/MS analyses revealed a striking lipid redistribution in bEVs: phosphatidylglycerol was significantly enriched, while cardiolipin was depleted compared to the cell membrane, supporting the notion that selective lipid sorting rather than nonspecific membrane shedding is involved in bEVs biogenesis (Biagini et al. 2015). Consistent with this, *S. pyogenes* bEVs were also enriched in lipoproteins which are known virulence factors in other pathogens. A similar pattern was observed in *S. pneumoniae*, where bEVs were enriched in lipoproteins and short-chain fatty acids relative to the parent membrane (Biagini et al. 2015). The shift toward unsaturated, short-chain acyl chains increases bilayer fluidity, promoting curvature and membrane vesicle release (Mercier et al. 2012). A regulatory layer to lipid remodeling is suggested by studies in *S. mutans* (Wang et al. 2025). Detailed lipidome comparisons of wild-type and an OpuB-deficient strain showed significant differences in bEVs phospholipid and fatty acid composition, which became more pronounced under acidic growth (pH 5.5). Acidification enhanced phospholipid differences, with OpuB mutant bEVs exhibiting greater increases in select fatty acid species (Wang et al. 2025). These findings support a role for OpuB in regulating lipid synthesis under acid stress, reinforcing membrane stability. Together with the proteomic changes observed in acidified *S. mutans* bEVs (Cao et al. 2020), these data highlight that pH adaptation is not confined to the cellular level. Rather, bEVs themselves may act as diffusible vehicles of stress adaptation, potentially influencing the broader microbial community by modulating both *S. mutans* physiology and the responses of neighboring species.

Streptococci are known to scavenge fatty acids and host-derived lipids. For example, the incorporation of phosphatidylcholine (Joyce et al. 2019), one of the most abundant and functionally important phospholipids in eukaryotic membranes, may influence both the production and stability of bEVs. Because phosphatidylcholine is electrically neutral yet polar, it readily supports the formation of stable bilayers (Van Meer et al. 2008). At the same time, its presence could help bEVs evade immune detection by mimicking host-derived components, a strategy that has been discussed to represent a form of rudimentary molecular mimicry (Joyce and Doran 2023). This is certainly an area of lipid vesicle membrane biology that requires further investigation with appropriate models that simulate the presence of host lipids. Metabolites are also crucial components of streptococcal bEVs. A recent exploration found 37 metabolites in *S. mutans* bEVs that were differentially expressed as a function of varying pH values (Cao et al. 2020), including betaine, linoleic acid, L-glutamate, erucamide, and palmitic acid, many of which are involved in fatty acid biosynthesis and linoleic acid metabolism. Combined proteomic and metabolomic analyses revealed pH-sensitive cargo changes, principally associated with ABC transporters, leading the authors to speculate that this pathway may be strongly implicated in streptococcal vesiculogenesis (Cao et al. 2020).

Together, these findings establish bEVs as selectively loaded, environmentally responsive delivery systems that support streptococcal adaptation and ecological success.

## bEVs in microbial Interactions and biofilm ecology

As previously discussed, the oral cavity is a multispecies ecosystem in which microbial survival depends upon a complex interplay of cell–cell signaling and numerous synergistic/antagonistic ecological interactions. Streptococcal bEVs contribute actively to this communication network, influencing both microbial components of the biofilm and host cells. Their impact on biofilm dynamics is particularly notable. bEVs from *S. mutans* enhance its own biofilm formation under sucrose-rich conditions, both in the presence and absence of saliva, thereby reinforcing its cariogenic potential (Wen et al. 2021). The crucial virulence gene *sfp* associates with bEVs vesiculogenesis in *S. mutans*. The *Δsfp* mutant strains exhibits changes in bEVs yield as well as susceptibilities to oxidative stress and low pH. At the same time, *S. mutans* bEVs inhibit the development of biofilms formed by commensal microbes—including *S. sanguinis* and *S. gordonii* (Wu et al. 2020, Cui et al. 2022), but did not impact planktonic growth. Additionally, expression of *gtfG, gtfP*, and *spxB* virulence factors was downregulated after exposure to *S. mutans* bEVs, demonstrating the central role of these vesicles in interspecies interactions in the context of initial surface colonization.

In contrast, *S. mutans* bEVs promote synergistic interactions with *Candida albicans*, promoting biofilm development and adhesion in a process mediated by vesicle-associated glycosyltransferases (Biswas and Biswas 2006, Cui et al. 2022, Wu et al. 2020). Interestingly, *S. mutans* bEVs were observed within the *C. albicans* EPS following co-incubation, which led to upregulation of crucial fungal transcriptional regulators including *Ndt80, Als1, Mnn9, Van1, Pmr1, Gca1*, and *Big1*. These findings suggest a strong association between bEVs-mediated interspecies and interkingdom communication during cariogenic dysbiosis. This promotion of biofilm growth and persistence may also play a key role in bacterial progression in periodontal disease pathogenesis, creating ecological niches that favor the late colonization of *P. gingivalis, F. nucleatum*, and other pathobionts (Abdulkareem et al. 2023).

Finally, streptococcal bEVs have also been found to participate in interactions with bacteriophages, adding yet another dimension to their physiological and pathological significance. For instance, *S. pyogenes* bEVs have been shown to bind to bacteriophage A1 and act as a decoy that protects the bacterial population from phage attack (Beerens et al. 2021). This occurs via vesicle-mediated scavenging of bacteriophages from the environment that was observed in multiple *S. pyogenes* strains. The bacteriophage-protective action of bEVs has also been shown for other bacteria including *Escherichia coli* and *Vibrio cholerae*, suggesting a conserved mechanism that protects microbes from phage attack (Reyes-Robles et al. 2018, Xuan et al. 2024). This defensive strategy underscores the versatility of bEVs-mediated processes, which encompass survival, ecological competition, host modulation, and protection against external threats. Taken together, these studies demonstrate that streptococcal bEVs are complex and active biological components within host-pathogen interactions and oral biofilm ecology, acting as “molecular messengers” that shape microbial function, influence host responses, and ultimately contribute to both health and disease.

## Host cell interactions and immune modulation by streptococcal bEVs

In the oral cavity, most microbial cells are embedded within biofilms and encased in an extracellular polymeric substance (EPS) matrix (Jakubovics et al. 2021), limiting the frequency and duration of direct contact with host cells or mucosal surfaces. A wide range of oral microbes, including both Gram-negatives [e.g. *P. gingivalis* (Haugsten et al. 2026)*, Fusobacterium nucleatum* (Engevik et al. 2021)] and Gram-positives [e.g. *Streptococcus* spp., *Corynebacterium* ssp. (Treerat et al. 2024), release bEVs (Leiva-Sabadini et al. 2025)]. It is plausible that vesicles serve as a primary means of host–microbe communication. Indeed, recent work demonstrates that bEVs can elicit immunological effects similar to those of intact bacterial cells (Helliwell et al. 2023), underscoring their direct impact on host physiology.

This also highlights a potentially underappreciated link between the microbial secretome and bEVs biology. Oral microbes are known to secrete a wide array of proteins, metabolites, and virulence factors, yet it remains unclear to what extent these secreted components are organized and trafficked within vesicles rather than being released individually. Since many experimental approaches used to study protein secretion also co-purify vesicles, distinguishing between free and vesicle-associated factors is often overlooked. Importantly, vesicular packaging not only protects labile cargo (Collins and Brown 2021) but, owing to the lipophilic nature of the membrane, may also facilitate efficient delivery and internalization into host cells (Mulcahy et al. 2014) or neighboring microbes (Tran and Boedicker 2017) amplifying their role as key mediators of intercellular communication in fact, it has been shown that *cif* toxin from *P. aeruginosa* is more effective when delivered via vesicles and interacts with the host’s epithelial cells (Bomberger et al. 2009).

### Internalization of bEVs by host cells

Internalization of streptococcal bEVs (Fig. 4A) has been demonstrated in several different host cell types. This phenomenon was first described for *S. pneumoniae*, where vesicles from the TIGR4 strain were shown to enter both A549 lung epithelial cells and human monocyte–derived dendritic cells (DCs) (Codemo et al. 2018). Internalization into DCs increased the abundance of MHC-II and CD86 on the cell surface, indicating DC activation, and was accompanied by cytokine release, including IL-6, IL-8, IL-10, and TNF-α (Tumor Necrosis Factor-α). The exposure of A549 cells to pneumococcal bEVs did not result in significant cytotoxicity. Similarly, primary human DCs readily internalized bEVs, eliciting only modest pneumolysin-dependent cell death, which was minor compared to the strong cytotoxic effect of intact TIGR4 bacteria. Hemolytic activity against erythrocytes was also observed in a dose-dependent manner and attributed to pneumolysin cargo, since bEVs isolated from a pneumolysin mutant lacked this activity. However, the hemolysis caused by bEVs was much lower than that of purified pneumolysin (Codemo et al. 2018). Collectively, these findings suggest that pneumococcal bEVs are primarily designed to promote an inflammatory environment without severe cytotoxic effects and thereby contribute to the pathology of invasive pneumococcal disease symptoms (Codemo et al. 2018).

**Figure 4:**
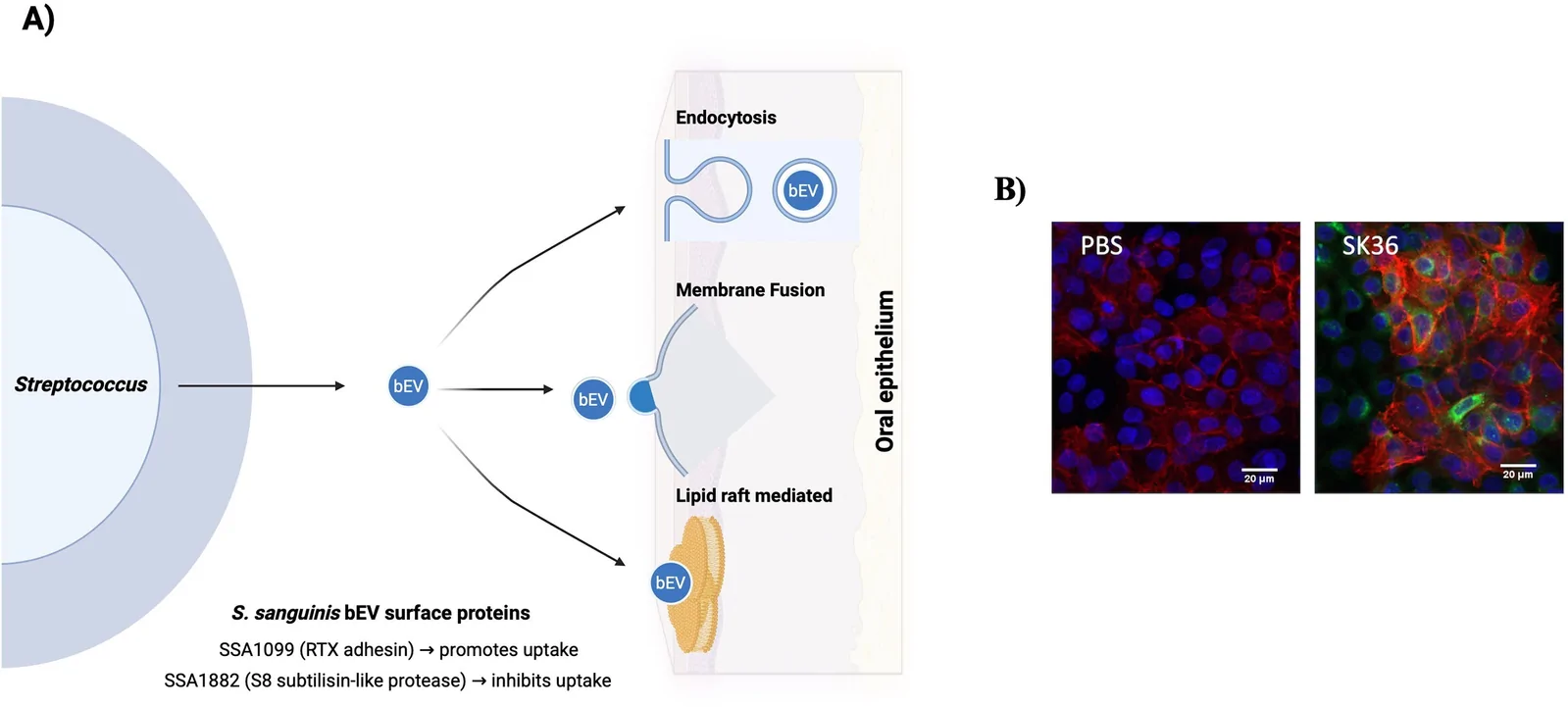
bEVs and their uptake by host cells. (A) Proposed routes of bEV uptake and host-cell interaction. Streptococcal bEVs can enter oral epithelial cells through multiple pathways, including endocytosis, direct membrane fusion, and lipid raft-mediated uptake. Following internalization, vesicle cargo can modulate host-cell signaling and gene expression. Uptake efficiency is influenced by vesicle-associated proteins. In *S. sanguinis*, the RTX adhesin and calcium-binding hemolysin-like protein SSA1099 promotes bEV internalization, whereas the predicted S8 subtilisin-like protease SSA1882 suppresses uptake, suggesting that vesicle surface proteins play important roles in regulating host–vesicle interactions. (B) Cellular internalization of *S. sanguinis* SK36 bEVs. Representative images of gingival epithelial cells (GECs) incubated with PBS (control) or SK36-derived bEVs. GEC nuclei are stained with DAPI, actin with Texas Red–phalloidin, and bEVs with DiO (Modified from Fig. 2A in reference: Helliwell et al. 2025; under CC-BY 4.0 license). (Figure generated using BioRender).

Macrophage internalization has also been investigated using bEVs derived from different pneumococcal serotypes. All serotype specific bEVs tested were readily internalized, but the resulting proinflammatory responses varied by serotype, as measured by cytokine release (Olaya-Abril et al. 2021). Internalization and subsequent inflammatory responses have been observed with other streptococci. For example, *S. pyogenes* bEVs were internalized by THP-1 cells differentiated into adherent macrophage-like cells (Murase et al. 2021), and *S. agalactiae* bEVs entered HeLa cells, resulting in a dose-dependent reduction in epithelial cell viability (Surve et al. 2016).

### Mechanisms of vesicle uptake

Bacterial membrane vesicles exploit multiple entry routes into eukaryotic cells, including clathrin- and caveolin-mediated endocytosis, macropinocytosis, phagocytosis, lipid raft–dependent pathways, and in some cases, direct fusion with host membranes, reviewed in (Mulcahy et al. 2014). These pathways have not been systematically investigated in streptococci. The clearest evidence for an endocytosis-based mechanism comes from *S. mutans*, where bEVs co-localized with endosomal markers (Oh et al. 2025). By contrast, direct fusion has been demonstrated for *S. aureus*, where bEVs deliver α-toxin to host cells in a cholesterol-dependent manner. Cholesterol sequestration by filipin III treatment blocked fusion, suggesting a lipid raft–associated, cholesterol-dependent mechanism for the tested HeLa cell line (Thay et al. 2013). Furthermore, Wang et al. reported that the internalization of *S. aureus* bEVs into human macrophages occurs primarily via a dynamin-dependent endocytic mechanism in order to deliver virulence molecules into host cells (Wang et al. 2020). Direct evidence for lipid raft–mediated bEVs uptake in streptococci is still missing; however, given its established role in Gram- negative and eukaryotic vesicle uptake (Furuta et al. 2009, O’Donoghue and Krachler 2016), this pathway may also be relevant for Gram-positives.

### Vesicle-associated factors influencing uptake

Confocal microscopy with the lipophilic dye Vybrant-DiO confirmed that *S. sanguinis* bEVs are efficiently taken up by oral epithelial cells, where they alter transcriptional activity and exert immunomodulatory effects (Helliwell et al. 2023) (Fig. 4B). Follow-up studies suggested that uptake of bEVs is at least partly dependent on vesicle-associated proteins. Proteomic and genetic analyses identified two key determinants with opposite roles. Deletion of SSA1099, an RTX adhesin and calcium-binding hemolysin-like protein, led to a marked reduction in bEVs internalization. Conversely, deletion of SSA1882, a predicted S8 subtilisin-like protease, resulted in increased bEVs entry, implying that this protease normally dampens uptake, possibly by modifying bEVs surface properties or interfering with host–vesicle interactions (Helliwell et al. 2025). Together, these findings underscore both the diversity of uptake routes and the species-specific regulatory factors that determine how streptococcal bEVs engage with host cells.

### Immune recognition and inflammatory response

The uptake of bEVs by host cells suggests that streptococcal bEVs may directly influence host–pathogen interactions from within. However, it is important to keep in mind that bEVs also carry pathogen-associated molecular patterns (PAMPs), such as lipoproteins, peptidoglycan fragments, and lipoteichoic acids which are all conserved microbial motifs recognized by pattern recognition receptors (PRRs) of the host immune system (Peregrino et al. 2024). These PAMPs can elicit immune responses without requiring internalization of the vesicles since most are associated with the plasma membrane (PRR like Toll-like receptors 1, 2, and others) (Takeuchi and Akira 2010). Experimentally distinguishing between immune activation caused by direct uptake versus receptor-mediated surface signaling remains challenging, and most likely both mechanisms occur simultaneously, resulting in a combined immune response (Fig. 5A).

**Figure 5:**
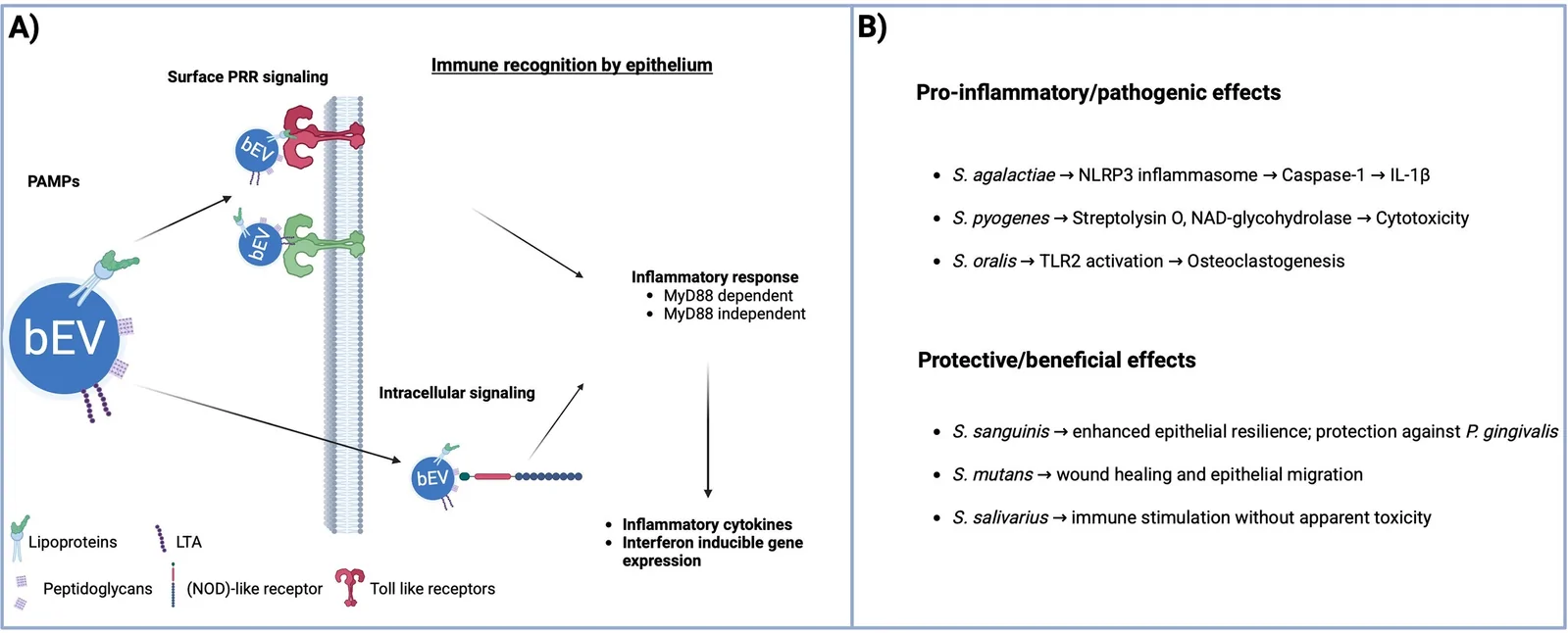
Mechanisms of streptococcal host-interaction and immune modulation. (A) Streptococcal bacteria release membrane vesicles containing proteins, nucleic acids, and other bioactive and immunogenic cargo such as lipoproteins, peptidoglycan fragments and LTA (lipoteichoic acid). Host cells recognize bEVs through Pathogen-Associated Molecular Patterns (PAMP)-mediated activation of Pattern-Recognition Receptors (PRR) such as TLR2 or TLR4 and through direct internalization and recognition by (NOD)-Like Receptors. TLR2 is the primary receptor for bacterial lipoproteins and LTA, However, TLR2 can form heterodimers with TLR1 to recognize mainly triacylated lipoproteins and with TLR6 to recognize mainly diacylated lipoproteins. (B) Host-interactions trigger diverse downstream responses ranging from cytokine production and inflammasome activation to tissue repair and protection against pathogenic microbes. The outcome depends on the streptococcal species, vesicle cargo, and host cell type. (Figure generated using BioRender).

This complexity raises an additional challenge when translating *in vitro* findings to the *in vivo* situation. In a living host, one can presume exposure not only to bEVs but also to host-derived exosomes. Therefore, bEVs are likely continuously present and maintain a baseline level of cytokine expression higher than that observed in cell culture grown without this constant challenge. Moreover, the mixture of vesicles *in vivo* from diverse microbial and host origins may elicit distinct or even synergistic/antagonistic responses compared to the isolated single-species vesicle systems typically used *in vitro*.

Despite these complexities, there is consistent evidence that bEVs trigger strong proinflammatory responses in host cells. For instance, a comparative study of *S. pneumoniae* and several *S. mitis* strains demonstrated robust cytokine induction in THP-1 macrophages after 4 h of exposure to purified bEVs (Matsumoto et al. 2025). The levels of IL-1β, IL-6, IL-8, IL-10, and TNF-α increased markedly, with IL-6 and IL-10 showing up to 10- to 100-fold increases. Interestingly, while TNF-α expression rose uniformly in THP-1 macrophages, HL-60 neutrophil-like cells failed to produce detectable levels of TNF-α under similar conditions (Matsumoto et al. 2025), indicating that cytokine responses to bEVs are cell-type specific and may reflect the distinct immunological roles of different host cells.

Unexpectedly, under the same experimental conditions, *S. sanguinis* SK36 did not produce detectable bEVs (Matsumoto et al. 2025), despite being known as a prolific vesicle producer capable of inducing IL-6 and IL-8 secretion in TIGK gingival epithelial cells (Helliwell et al. 2025). A more detailed study investigating the signaling pathways triggered by *S. agalactiae* bEVs revealed that vesicles from invasive strains strongly activated caspase-1 via the NLRP3 inflammasome in THP-1 cells, as inhibition of NLRP3 abrogated caspase-1 activity and IL-1β processing (McCutcheon et al. 2025). Given that *S. agalactiae* is an opportunistic pathogen associated with severe neonatal infections, these findings suggest that bEVs play a key role in promoting inflammation and tissue damage—consistent with the pathology of *S. agalactiae*-mediated chorioamnionitis, an infection of the amniotic sac and placenta that can lead to preterm birth, fetal death, and brain injury (McCutcheon et al. 2025).

Interestingly, not all bEVs trigger tissue damage through inflammatory activation and increased cytokine abundance was not necessarily associated with cell death. For example, it has been shown that *S. mutans* bEVs stimulate epithelial cell proliferation and migration in oral mucosal organoids, while also accelerating wound healing in murine skin models Oh et al. 2025), mostly via tRNA-mediated communication and TLR-3 dependent mechanisms. bEVs from the commensal *S. salivarius*, which carried 466 proteins associated with metabolism and cell wall arrangement, were found to induce interleukin production in human THP-1 monocytic cells, but when tested in a *Galleria mellonella* infection model showed no obvious toxicity (Kulig et al. 2023b). Similarly, bEVs from *S. sanguinis* can be taken up by gingival keratinocytes (Helliwell et al. 2025). Following coincubation with bEVs, a downregulation of interferon signaling and apoptosis-related genes, coupled with an upregulation of genes involved in cytokine production was observed. Most interestingly, pre-exposure of gingival epithelial cells to bEVs from *S. sanguinis* protected them from *P. gingivalis*-associated dissociation and cellular death, suggesting a protective commensal role of *S. sanguinis* against tissue destruction by periodontal pathogens. *S. pyogenes* has also been shown to generate bEVs with the ability to provoke robust cytokine responses in monocytic cell lines, aligning with their well-known pro-inflammatory pathogenic profile (Murase et al. 2021). *Streptococcus pyogenes* bEVs were found to contain important virulence proteins such as streptolysin O and NAD-glycohydrolase, which can be directly delivered to host cells via vesicle-based communication. Strengthening their connection with pathogenesis, bEVs derived from invasive strains were more abundant and showed much higher cytotoxicity and cytokine expression in target cells (Murase et al. 2021). Another study elucidated the role of *S. oralis* bEVs on osteoclast activity. Its vesicles were found to activate TLR2 pathways thereby promoting osteoclastogenesis (Kim et al. 2022), highlighting the potential virulence that oral commensal bacteria can have outside of their normal ecological niche. Collectively, these findings demonstrate that streptococcal bEVs exert dual roles, by influencing microbial behavior as well as modulating host tissues and immune responses (Fig. 5B).

## Potential applications and therapeutic perspectives

Owing to their direct implication in health and pathogenesis of oral diseases, there is a newly heightened interest in the diagnostic, preventive, and therapeutic use of oral streptococci derived bEVs. As these vesicles are known to reflect the metabolic state of bacteria or environmental conditions, they represent highly recognizable molecular “fingerprints” that could be used as biomarkers in saliva or gingival crevicular fluid to predict disease development. Thus, oral bacteria bEVs are readily available for non-invasive sampling and could easily be detected with point-of-care diagnostic tests in the clinical practice. The development of novel molecular assays for detecting bEVs in complex oral fluids is also an exciting potential avenue to be explored in the near future (Contreras-Naranjo et al. 2017, Reátegui et al. 2018, Boulestreau et al. 2024).

There is also increasing interest in the therapeutic utility of bEVs as postbiotics, nonviable bacterial-derived components (Vinderola et al. 2022), to modulate local biofilm dysbiosis and host immune responses. For example, utilizing bEVs from oral commensal strains such as *S. sanguinis* for controlled delivery to the oral mucosa could help prevent invasion by inflammatory pathobionts (Helliwell et al. 2025) and aid in periodontal health maintenance in high-risk individuals. As streptococcal vesicles are naturally immunogenic, they could be repurposed for antigen presentation or drug delivery as “nanovaccines”. In this regard, there are some proof-of-concept studies with *S. pneumoniae* bEVs that were able to protect mice from pneumococcal infection (Choi et al. 2017), and there is promise that these treatments could also be developed for oral biofilm-mediated diseases. Their nanoscale size, bioactive cargo, and ability to be quickly incorporated into host cells with functional results suggest they could be excellent candidates for drug delivery applications (Liu et al. 2022). Furthermore, the possibility of artificially loading EVs with drugs of interest (Carrasco-Rojas et al. 2025) or antimicrobial peptides to facilitate their targeted delivery and release opens a myriad of possibilities to utilize oral bEVs as nanocarriers against pathogens. The successful implementation of OMV-based vaccines derived from *Neisseria meningitidis* serogroup B demonstrates the feasibility of vesicle-based vaccination strategies. Although these licensed vaccines consist of detergent-extracted and extensively processed OMVs rather than native vesicles, they have proven both safe and effective. Furthermore, vaccination with serogroup B OMV vaccines has been associated with partial protection against *N. gonorrhoeae*, likely due to shared surface antigens between the two species (Petousis-Harris et al. 2017). Together, these findings provide clinical proof of principle that bacterial vesicles can function as effective antigen delivery systems and highlight the potential of engineered bEVs for the development of future vaccines.

Several key challenges remain to implement bEVs-based technologies for dental and biomedical applications. Firstly, it is well known that EVs can have important variations as a function of the bacterial strain, environmental conditions, and employed EV isolation method (Suresh and Zhang 2025). Secondly, saliva is a highly complex and diverse matrix containing a combination of EVs from microbial and host origins, which may hinder the isolation of strain-specific bEVs in the clinical setting. Furthermore, certain bEV isolation methods are incompatible with good manufacturing practices (GMP) standard (Cui et al. 2024, Humbert et al. 2025, Wiest and Zubair 2025). Thus, important field-wide standardizations for streptococcal EV isolation and normalization (i.e. strain characteristics, growth phase), dosing normalizations (i.e, particles per µg protein), and quality control must all be developed and optimized before bEVs-based therapeutics can be realized. Furthermore, the in-vitro effects of streptococal bEVs still must be demonstrated with robust in vivo and clinical studies that support translation of these findings into clinical practice. Resolving these challenges will be key in order to quickly and effectively deliver bEVs-based treatments against oral diseases to patients in a reproducible and predictable manner.
